# Membership and Feedback on the American Academy of Orthopaedic Surgeons and Other Subspecialty Societies: A Survey Study of Orthopaedic Surgeons

**DOI:** 10.5435/JAAOSGlobal-D-22-00226

**Published:** 2022-11-03

**Authors:** Arjun Saxena, Gregory R. Toci, Parker L. Brush, Alexis Reinhardt, Pedro K. Beredjiklian, Alan S. Hilibrand, Alexander R. Vaccaro, Daniel Fletcher

**Affiliations:** From the Rothman Orthopaedic Institute at Thomas Jefferson University Hospital, Philadelphia PA.

## Abstract

Orthopaedic surgeons face decreased reimbursement, lower income, and increased rates of burnout. As subspecializing through fellowship training in orthopaedics becomes more and more prevalent, the value of membership to a general orthopaedic society (American Academy of Orthopaedic Surgeons [AAOS]) warrants investigation.

**Methods:**

One hundred thirty orthopaedic surgeons were surveyed by e-mail through a 14-item anonymous survey administered through SurveyMonkey. The survey inquired about surgeon experience, practice type, fellowship training, and details regarding AAOS and subspecialty society membership.

**Results:**

The response rate was 67%, with 94% of respondents indicating that they were members of AAOS and a subspecialty society. The most common reasons for AAOS membership were tradition (65, 74.7%), continuing medical education (46, 52.9%), maintenance of board certification (44, 50.6%), and political advocacy (40, 46.0%). The most common reasons for subspecialty society membership were continuing medical education (73, 83.9%), tradition (49, 59.8%), and political advocacy (33, 40.2%).

**Discussion:**

Most surgeons in our study cohort were members of both AAOS and a subspecialty society, but the reasons for membership in each differed. Almost 80% of respondents think their subspecialty society provides all their professional needs. The orthopaedic societies need to continue to evolve to provide value to their members to succeed in the future.

The American Academy of Orthopaedic Surgeons (AAOS) was founded in 1933 at Northwestern University. Currently based in Rosemont, Illinois, the Academy provides musculoskeletal education to patients, orthopaedic surgeons and allied health professionals, and orthopaedic surgeons throughout the world. The American Association of Orthopaedic Surgeons, founded by the Academy board of directors in 1997, engages in health policy and advocacy activities for patients and providers of orthopaedic surgery. Although the Academy and the Association are two separate but related entities, they are both commonly referred to as the AAOS. The mission of the AAOS is to serve the orthopaedic profession to provide the highest-quality musculoskeletal care with three strategic goals including experience, quality, and culture.

The first orthopaedic subspecialty society was the American Society for Surgery of the Hand, which was established in 1946. More recently, the emergence of specialty societies and focus on subspecialties in orthopaedics have changed educational and participation patterns for all orthopaedic surgeons. As orthopaedics has become more specialized, general educational opportunities, advocacy, and member benefits from the AAOS may not be as valued as opportunities from within their respective subspecialty societies.

Orthopaedic surgeons are facing decreased reimbursements and lower incomes as well as increased pressure and rates of burnout at work. Therefore, the value of membership to both general and subspecialty societies is worth investigating because they incur notable financial dues and time commitments away from work and personal life to attend conferences and meetings. Furthermore, as subspecializing through fellowship training in orthopaedics becomes more and more prevalent, the value of membership to a general orthopaedic society (such as AAOS) also warrants investigation. We hypothesized that the subspecialization of orthopaedic surgeons and escalating cost of society membership have led to decreased membership and decreased perceived value of the AAOS. Thus, we conducted a survey study to assess the perceived value of AAOS and subspecialty society membership among a cohort of orthopaedic surgeons.

## Methods

**Figure 1:**
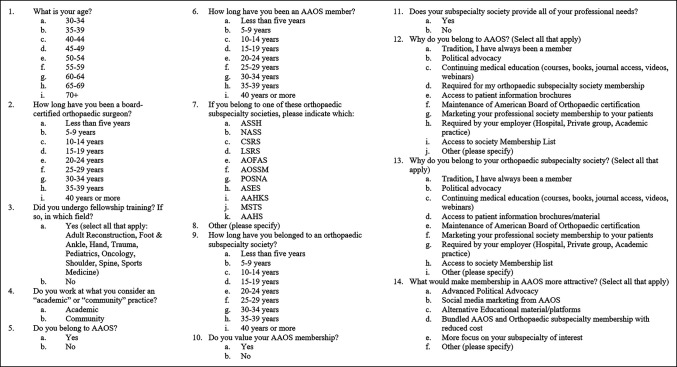
Diagram showing survey details. AAHS = American Association for Hand Surgery, AAHKS = American Association of Hip and Knee Surgeons, AOFAS = American Orthopaedic Foot and Ankle Society, AOSSM = American Orthopaedic Society of Sports Medicine, ASES = American Shoulder and Elbow Surgeons, ASSH = American Society for Surgery of the Hand, CSRS = Cervical Spine Research Society, LSRS = Lumbar Spine Research Society, NASS = North American Spine Society, POSNA = Pediatric Orthopaedic Society of North America, MSTS = Musculoskeletal Tumor Society

All orthopaedic surgeons at one large orthopaedic surgery group and all orthopaedic surgeons on staff at a community medical center were surveyed through e-mail. The combined orthopaedic cohort consisted of 130 licensed orthopaedic surgeons across four states, primarily located in the Northeastern United States. This cohort consisted of 18 (13.8%) surgeons specialized in spine surgery, 29 (22.3%) in arthroplasty, 21 (16.2%) in hand and wrist, 11 (8.5%) in foot and ankle, 8 (6.2%) in shoulder and elbow, 7 (5.4%) in traumatology, 2 (1.5%) in oncology, 24 (18.5%) in sports medicine, 2 (1.5%) in pediatrics, and 8 (6.2%) in general orthopaedics. A survey reminder was sent two weeks after the initial e-mail, and the survey was open for one month. The survey was a 14-item anonymous survey through SurveyMonkey. The questions were formulated to inquire about surgeon experience, practice type, and fellowship training as well as details regarding AAOS and orthopaedic subspecialty society membership. Full details regarding the questionnaire are presented in Figure 1. All responses were categorical, and data were reported as the sample size with corresponding percentages. AAOS and subspecialty society membership fees were collected online from public websites or from members.

## Results

**Figure 2:**
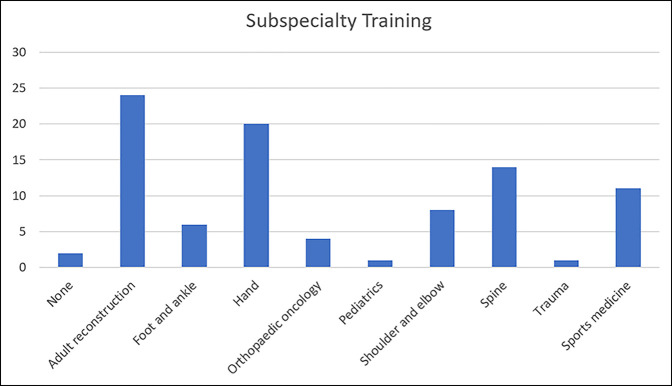
Graph showing fellowship training of survey population.

Of the 130 surgeons who received the survey, 87 responded (response rate: 67%). Fifty-nine surgeons (67.8%) considered their practice to be academic while 23 surgeons (26.4%) considered their practice to be community. The remaining five surgeons did not specify whether their practice was academic or community. The most common age was 45 to 49 years (19, 21.8%), and the most common level of experience was 10 to 14 years (20, 23.0%). All orthopaedic fellowships were represented, and the most common was adult reconstruction (24, 27.6%) (Figure 2). Two (2.30%) respondents did not complete fellowship training (Table 1).

**Table 1:** Surgeon Characteristics

Total N	87
Type of practice	
Academic	59 (67.8%)
Community	23 (26.4%)
Age, yr	
35-39	10 (11.5%)
40-44	12 (13.8%)
45-49	19 (21.8%)
50-54	11 (12.6%)
55-59	16 (18.4%)
60-64	7 (8.05%)
65-69	5 (5.75%)
≥70	2 (2.30%)
Experience, yr	
<5 yr	13 (14.9%)
5-9 yr	10 (11.5%)
10-14 yr	20 (23.0%)
15-19 yr	12 (13.8%)
20-24 yr	16 (18.4%)
25-29 yr	5 (5.75%)
30-34 yr	8 (9.20%)
35-39 yr	0 (0%)
≥40 y	3 (3.45%)
Fellowship training	
None	2 (2.30%)
Adult reconstruction	24 (27.6%)
Foot and ankle	6 (6.90%)
Hand	20 (23.0%)
Orthopaedic oncology	4 (4.60%)
Pediatrics	1 (1.15%)
Shoulder and elbow	8 (9.20%)
Spine	14 (16.1%)
Trauma	1 (1.15%)
Sports medicine	11 (12.6%)

Of the 87 respondents, 82 (94.3%) indicated that they were currently members of the AAOS. Five (5.75%) surgeons of varying levels of experience indicated that they were not currently members, of whom two were fellowship-trained hand surgeons and three were spine surgeons with subspecialty membership in the American Society for Surgery of the Hand and the North American Spine Society and the Cervical Spine Research Society, respectively. The most common membership duration was 20 to 24 years (18, 20.7%). Sixty-four (73.6%) respondents valued their AAOS membership while 23 (26.4%) did not, including five hand surgeons, nine adult reconstruction surgeons, two foot and ankle surgeons, six spine surgeons, and one sports medicine surgeon. The most commonly selected reasons for belonging to AAOS were tradition (65, 74.7%), continuing medical education (46, 52.9%), maintenance of board certification (44, 50.6%), and political advocacy (40, 46.0%). The most commonly selected option for improving AAOS membership was bundling AAOS and subspecialty society memberships (50, 57.5%), followed by advanced political advocacy (25, 28.7%), alternative educational materials and platforms (24, 27.6%), and more focus on the respondent's subspecialty of interest (24, 27.6%) (Table 2).

**Table 2:** AAOS Membership

Total N	87
Members	82 (94.3%)
Length of membership (years)	
<5	3 (3.45%)
5-9	10 (11.5%)
10-14	19 (21.8%)
15-19	15 (17.2%)
20-24	18 (20.7%)
25-29	5 (5.75%)
30-34	9 (10.3%)
35-39	0 (0%)
≥40	3 (3.45%)
Value AAOS membership	64 (73.6%)
Reasons for membership	
Tradition	65 (74.7%)
Political advocacy	40 (46.0%)
Continuing medical education	46 (52.9%)
Required for subspecialty society	9 (10.3%)
Access to patient information brochures	4 (4.60%)
Maintenance of board certification	44 (50.6%)
Marketing membership to patients	10 (11.5%)
Required by employer	2 (2.30%)
More attractive AAOS membership	
Advanced political advocacy	25 (28.7%)
Social media marketing from AAOS	12 (13.8%)
Alternative educational material	24 (27.6%)
Bundled AAOS and subspecialty society membership	50 (57.5%)
More focus on subspecialty of interest	24 (27.6%)

AAOS = American Academy of Orthopaedic Surgeons

**Figure 3:**
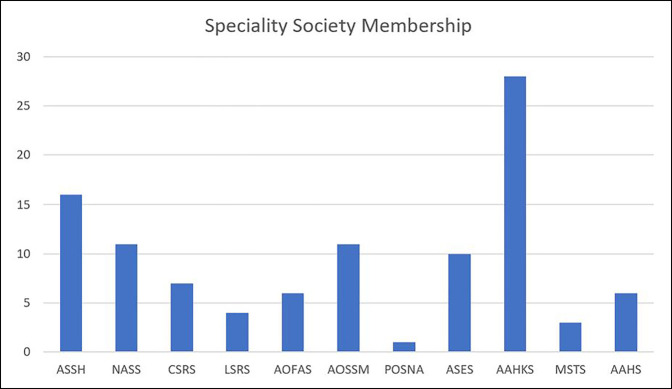
Graph showing representative subspecialty membership of the survey population. AAHS = American Association for Hand Surgery, AAHKS = American Association of Hip and Knee Surgeons, AOFAS = American Orthopaedic Foot and Ankle Society, AOSSM = American Orthopaedic Society of Sports Medicine, ASES = American Shoulder and Elbow Surgeons, ASSH = American Society for Surgery of the Hand, CSRS = Cervical Spine Research Society, LSRS = Lumbar Spine Research Society, NASS = North American Spine Society, POSNA = Pediatric Orthopaedic Society of North America, MSTS = Musculoskeletal Tumor Society

Of the 87 respondents, 5 (5.75%) indicated that they were not members of an orthopaedic subspecialty society and 19 (21.8%) indicated that they were members of more than one orthopaedic subspecialty society. Three of these surgeons were fellowship-trained hand surgeons and had been members of AAOS for 20 to 24 years, whereas the other two surgeons were fellowship-trained spine surgeons who were also not members of AAOS. Eighteen different subspecialty societies were represented, with the most common being the American Association of Hip and Knee Surgeons (28, 32.2% total and 100.0% of fellowship-trained adult reconstruction surgeons) and the American Society for Surgery of the Hand (16, 18.4% total and 80.0% of fellowship-trained hand surgeons) (Figure 3). The most common duration of subspecialty society membership was 10 to 14 years (18, 20.7%), and 69 respondents (79.3%) stated that their subspecialty society provides all their professional needs. The most common reasons for subspecialty society membership were continuing medical education (73, 83.9%), tradition (49, 59.8%), and political advocacy (33, 40.2%) (Table 3). Membership fees for AAOS and subspecialty societies are summarized in Table 4.

**Table 3:** Subspecialty Society Membership

Total N	87
No subspecialty society membership	5 (5.75%)
Longest subspecialty membership (yr)	
<5	8 (9.20%)
5-9	11 (12.6%)
10-14	18 (20.7%)
15-19	16 (18.4%)
20-24	13 (14.9%)
25-29	6 (6.90%)
30-34	5 (5.75%)
35-39	0 (0%)
≥40	0 (0%)
Subspecialty society provide all professional needs	69 (79.3%)
Reasons for membership	
Tradition	49 (56.3%)
Political advocacy	33 (37.9%)
Continuing medical education	73 (83.9%)
Access to patient information brochures	15 (17.2%)
Maintenance of board certification	18 (20.7%)
Marketing membership to patients	18 (20.7%)
Required by employer	2 (6.90%)

**Table 4:** Annual Membership Fees

Organization	Membership fee ($)
American Academy of Orthopaedic Surgeons^1^	1055
North American Spine Society^2^	655
Cervical Spine Research Society^3^	950
Scoliosis Research Society^4^	575
International Society for the Study of the Lumbar Spine^5^	150
International Society for Advancement Of Spine Surgery^6^	295
American Society for Surgery of the Hand^7^	750
Orthopaedic Trauma Association^8^	800
American Orthopaedic Society for Sports Medicine^9^	750
American Orthopaedic Foot and Ankle Society^10^	625
American Association of Hip and Knee Surgeons^11^	600
Pediatric Orthopaedic Society of North America^12^	550
American Association for Hand Surgery^13^	440
New Jersey Orthopaedic Society^14^	300
Pennsylvania Orthopaedic Society^15^	500
American Shoulder and Elbow Surgeons^16^	800
Musculoskeletal Tumor Society^17^	450
Hip Society^18^	1500
Knee Society^19^	1500
Society for Minimally Invasive Spine Surgery^20^	295

## Discussion

Most of the orthopaedic surgeons in our study sample were members of the AAOS and a subspecialty society (both 94%). Our cohort consisted primarily of academic orthopaedic surgeons, and almost all had obtained specialized fellowship training. Most of the members valued their AAOS membership, and while there was notable variability in the reasons they valued their membership, the most commonly cited reasons were tradition, continuing medical education, maintenance of board certification, and political advocacy. Although these were also cited as reasons for subspecialty membership, the predominant reason was continuing medical education, and tradition and political advocacy were less frequently cited. Most of the respondents noted that bundled AAOS and subspecialty membership would increase the attractiveness of their AAOS membership, and other common answers included more political advocacy, more focus on the subspecialty of interest, and alternative educational materials.

AAOS delivers a personalized and seamless member experience while equipping members to thrive in a value-based environment and advance the quality of orthopaedic care. AAOS strives to evolve the culture and governance of AAOS's board and volunteer structure to become more strategic, innovative, and diverse. To achieve these strategic goals, the AAOS has engaged the following strategic enablers including advocacy, communication, partnerships, and technology.^22^ The goal of advocacy is to advance access to and quality of musculoskeletal health care and support providers to thrive in an evolving healthcare environment. AAOS plans to communicate renewed member value stemming from the new strategic plan. The vision includes partnerships that develop the right content, programs, and platforms to increase member value and drive greater effect. Finally, technology will be used to modernize platforms and offer seamless experiences. ^22,23^

AAOS membership costs $1,055, which may represent a notable financial burden for young academic orthopaedic surgeons. This is particularly true when considering subspecialty societies and their associated costs. In our study, one respondent was a member of not only AAOS but also the North American Spine Society, the Cervical Spine Research Society, the Scoliosis Research Society, the International Society for the Study of the Lumbar Spine, and the International Society for the Advancement of Spine Surgery, all of which totals up to $3680 in annual membership fees ($1055 for AAOS and up to $655, $950, $575, $150, and $295 for each subspecialty society, respectively). Furthermore, subspecialty societies are typically less expensive than AAOS (Table 4), but 80% of respondents stated that their subspecialty society provides all their professional needs. Many orthopaedic surgeons also support and are members of their local and state organizations at additional cost. For example, the annual due of the New Jersey Orthopaedic Society is $300 per year while the annual due of the Pennsylvania Orthopaedic Society is $500. As physician expenses continue to escalate while revenues decline, ancillary membership in AAOS and nonessential subspecialty societies may decline as well. Orthopaedic societies are competing for the same individuals for membership while they may benefit by bundling or combining memberships and society benefits.

We identified several publications regarding membership in orthopaedic societies.^21,24,25 26 27 28 29 30 31 32,33^ All these studies describe the importance of promoting diversity and inclusion within orthopaedic societies and orthopaedic leadership with exception to one study that describes the geographic distribution of their members.^27^ Scerpella et al. described the creation of the Forum to support women in orthopaedics and promote research endeavors. They also identified advocacy as an important area for members.^25^ Thevendran et al.^26^ further offered reasons to joint orthopaedic societies as opportunities for networking, education, research, mentorship, leadership, and career advancement. Specifically, authors have cited career advancement as an important reason to participate in subspecialty societies.^29,33^ These studies may describe reasons to join orthopaedic societies, but none have provided membership data on the value of membership. Unique to our study, we collected information from members themselves regarding reasons for joining orthopaedic societies. In contrast to prior literature, our data suggest that tradition and educational opportunities play key roles in societal membership.

There are limitations to this study. We were unable to survey all AAOS members or subspecialty societies because of difficulty in access to member contact lists. Although this approach would improve representation of AAOS and subspecialty society members, practicing orthopaedic surgeons without membership to either society would not be represented. Thus, we surveyed all orthopaedic surgeons at a large orthopaedic surgery group and all orthopaedic surgeons on staff at an associated community medical center, although this sample may be subject to regional and practice-specific biases. However, to the best of our knowledge, this represents the first study examining society membership in orthopaedic surgery, and responses from our 87 participants may provide useful feedback toward improving AAOS and subspecialty society groups. As the first study to research this topic, we hope to provide direction for the AAOS and subspecialty societies on how to better engage with their current members and potential members, but additional research is required to make recommendations.

In conclusion, most of the surgeons in our study cohort were members of both AAOS and a subspecialty society, but the reasons for membership in each differed among participants. Almost 80% of responding orthopaedic surgeons think that their subspecialty society provides all their professional needs. The AAOS and subspecialty societies need to continue to evolve to provide value to their members to succeed in the future.
